# A Cross-Sectional Study on Patient Satisfaction With Healthcare Services Provided at the Ophthalmology Clinics in Saudi Arabia

**DOI:** 10.7759/cureus.52202

**Published:** 2024-01-13

**Authors:** Asayel T Alruwais, Adhwaa Allihyani, Enas A Sindy, Raghad Alhowaidi, Oyoon Mulla, Jaylan S Malibari, Taif N Alhothali, Rawan Aljuwaybiri, Abdullah Alghamdi, Safa H Alkalash

**Affiliations:** 1 Medicine, Umm Al-Qura University, Makkah, SAU; 2 Obstetrics and Gynecology, King Abdulaziz University, Jeddah, SAU; 3 Ophthalmology, Umm Al-Qura University, Makkah, SAU; 4 Community Medicine and Health Care, Umm Al-Qura University, Al Qunfudhah, SAU; 5 Family Medicine, Menoufia University, Shebin Elkom, EGY

**Keywords:** healthcare customer satisfaction, saudi arabia, satisfaction, ophthalmology clinics, blindness, vision

## Abstract

Background

Saudi Vision 2030 is transforming the country's healthcare system, with efficacy, accessibility, and patient satisfaction with healthcare services serving as key indicators for assessing patient care quality. As blindness and impaired vision continue to be a rising health issue in most Eastern Mediterranean Region (EMR) nations, including Saudi Arabia, this study focused on measuring patient satisfaction with healthcare services in ophthalmology clinics.

Objectives

This study aimed to assess the level of patient satisfaction with healthcare services in ophthalmology clinics and its related factors in the Makkah region of Saudi Arabia in 2022-2023.

Methods

A cross-sectional study was conducted on a convenience sample of 553 Saudi and non-Saudi patients, aged 18 years and older, who attended private and government ophthalmology clinics in the Makkah region of Saudi Arabia during the period between November 2022 and February 2023. A self-administered questionnaire was distributed on several electronic platforms like WhatsApp, X (formerly known as Twitter), Telegram, and Snapchat to collect the data. Finally, all the data were entered and analyzed through the IBM SPSS software version 26.

Results

A total of 553 responses were obtained. The majority were aged between 18 and 35 years old (76.5%, n=423), with the female gender being dominant (79.7%, n=441). More than half of them (52.3%, n=289) preferred to receive ophthalmological healthcare services from governmental hospitals. The most commonly diagnosed eye disease was refractive error (43.2%, n=239). Patient satisfaction with healthcare services provided in ophthalmology clinics represented 75% (n=415). The odds of being satisfied with ophthalmology clinics are expected to decrease by at least 44% among individuals over the age of 35 (odds ratio (OR) =0.437; 95% CI=0.257-0.743; p=0.002). Moreover, those who had been married were predicted to decrease the chance of being satisfied by at least 50% compared to patients who had never been married (OR=0.538; 95% CI=0.352-0.823; p=0.004). Compared to students, patients who were currently employed were predicted to decrease the chance of being satisfied by at least 48% (OR = 0.481; 95% CI=0.270-0.856; p=0.013). Additionally, those with a higher monthly income had decreased odds of being satisfied by at least 58% (OR=0.583; 95% CI=0.381-0.893; p=0.013). In contrast, compared to patients with associated comorbidity, patients who have no comorbidity were predicted to have an increased chance of being satisfied by at least two-fold than those who had comorbidities (OR=2.023; 95% CI=1.199-3.413; p=0.008).

Conclusions

The study concludes that 75% of the patients attending ophthalmology clinics in the Makkah region of Saudi Arabia were satisfied with the healthcare services provided in these clinics. Most patients acknowledged the time of care, doctors' professionalism, continuity of care, comprehensive examination, and their education about their disease and management, in addition to doctors listening to them during their visits to ophthalmology clinics. Factors affecting patient satisfaction with medical services in ophthalmology clinics are patient age, occupation, marital status, monthly income, and associated comorbidities. Further studies are recommended to deeply understand patients' needs and obtain more suggestions to be fully satisfied with healthcare services in ophthalmology clinics and other different healthcare facilities.

## Introduction

One of the key targets of Saudi Vision 2023 is the Health Sector Transformation Program. It aspires to improve citizens' access to free health and healthcare services and insurance by providing equitable and comprehensive geographical coverage across the Kingdom of Saudi Arabia, extending electronic health services and digital technologies, and enhancing healthcare quality. Furthermore, the initiative aims to improve beneficiary satisfaction by integrating value-based healthcare and worldwide best practices and raising community awareness of road safety [1].

Patient satisfaction is a measure of the extent to which a patient is satisfied with their healthcare provider [2]. It has become a strategic variable and an important indicator of long-term viability and success by enhancing the outcomes of healthcare and the use of services [2]. The reasons for the dissatisfaction must be recognized and eliminated to enhance the quality of the care provided [3]. In the healthcare delivery sector, patient satisfaction surveys have been utilized to achieve three basic goals [3]. First, they assess how the patient’s decisions are affected by his or her satisfaction with the services provided. As a result, he or she will adhere to the treatment required and make ongoing use of care when needed. Second, patient satisfaction has been considered an important factor in increasing the quality of healthcare outcomes. Third, the surveys contribute to understanding the patient's needs, raising liability, and enhancing the services produced [3].

In recent years, eye diseases have become a serious public health concern, with major negative effects on human health, productivity, and the economy for patients, families, and the community [4]. At least 2.2 billion people worldwide suffer from near- or distance-impaired vision, and at least one billion of these people suffer from a visual impairment that could have been prevented or is still unaddressed [5]. In Saudi Arabia, a study shows that refractive errors were the primary source of visual impairment in 68 (24.5%) of the people. Trichiasis (1.8%, n=5), glaucoma (0.7%, n=2), and cataracts (7.2%, n=20) were some additional causes of visual impairment [6]. However, most vision impairments are preventable, treatable, or curable [4].

Many studies all over the world focused on the assessment of patient satisfaction with healthcare services. An Indonesian study in 2019 revealed that to improve patient satisfaction, alternative funding options for those without health insurance should be provided [7]. The waiting period for the examination is one of the main determinants of patient satisfaction with healthcare services [8-9]. Previous studies in ophthalmology and other settings revealed that technical quality, accessibility of care, and communication between doctors and their patients have played a role in patient satisfaction with healthcare services [10-14]. Other determinants of patient satisfaction with healthcare services include their ages, educational backgrounds, and income [15-16]. There was no previous research investigating patient satisfaction with healthcare services provided at the ophthalmology clinics in the Makkah region of Saudi Arabia; therefore, this study was done to assess the level of patient satisfaction with these services and its related factors as a step toward quality improvement.

## Materials and methods

Study overview

This cross-sectional study was conducted to assess the level and factors affecting patient satisfaction with the healthcare services provided in the ophthalmology clinics in the Makkah region of Saudi Arabia. The study was carried out after approval from the Biomedical Research Ethics Committee of Umm Al-Qura University, Makkah, Saudi Arabia (approval number: HAPO-02-K-012-2022-11-1295). Informed consent was obtained from each participant prior to filling out the questionnaire through a header that displays the purpose of the study, ensuring that all research data would be handled anonymously and that they were volunteers to accept or refuse participation.

Study criteria

The study included Saudi and non-Saudi patients, aged 18 years and older, who attended private and government ophthalmology clinics in the Makkah region of Saudi Arabia, between November 2022 and February 2023 and gave their consent to participate in the survey.

Study procedure

The main research data have been collected using a self-administered survey that was adopted from another Saudi study [12] and created as a Google Form (Google Inc., Mountainview, CA) in Arabic, which is the native language of Saudi Arabia. The electronic link to the survey was circulated on several electronic platforms, like WhatsApp, X (previously known as Twitter), Telegram, and Snapchat, among the target population for a period of four months, from November 2022 to February 2023. The total number of questionnaires completed, as determined by analyzing the data assembled, was 553. There were no unanswered questions.

Assessments

The utilized questionnaire consisted of two partitions; the first involved specific questions about sociodemographic characteristics (age, gender, marital status, occupation, education, and income). The second part of this survey consisted of 36 closed-ended questions and was subdivided into six dimensions of care. Each dimension has several statements that measure patient satisfaction: accessibility (four items), continuity (three items), humanness (six items), comprehensiveness (five items), communication (five items), education (10 items), and center satisfaction (three items).

Sample size

The minimum sample size required for this study was calculated using the Raosoftsample size calculator (2004, Raosoft Inc., Seattle, WA) in consideration of the following: the population size of Makkah region (85,57,766) [17], keeping the confidence interval (CI) level at 95%, the margin of error at 5%, and considering the anticipated percentage of satisfaction frequency at 50%. The minimum sample size was calculated to be at least 384 participants. However, we have received a total of 553 responses, which is more than the minimum calculated sample to overcome the issues of non-response or incomplete submissions and to help generalize the extracted findings because a large sample is more representative of the population.

Statistical analysis

The data were analyzed using the software program IBM Statistical Packages for Software Sciences (SPSS) version 26 (IBM Corp., Armonk, NY). For the descriptive analysis, the mean ± SD was used for quantitative variables, while numbers and percentages (%) were given for categorical variables. The total satisfaction of the patients regarding healthcare services provided in the ophthalmology clinics has been assessed using a three-point Likert scale category ranging from disagree, which was coded with "one," to agree, which was coded with "three" as the answer options. The total satisfaction score has been calculated by summation of all 36 items, and a score range of 36-108 has been generated. By using 75% as a cutoff point to determine the level of satisfaction, patients were considered dissatisfied if the score was below 75%, while those at and above 75% were categorized as satisfied. The differences in the level of satisfaction according to the socio-demographic characteristics were assessed using the Chi-square test. Significant results were then tested in a binary regression model to determine the independent significant predictors of satisfaction with corresponding odds ratios as well as 95% confidence intervals. Values with a p-value less than 0.05 are regarded as significant.

## Results

In total, 553 patients responded to this survey. The majority were aged between 18 and 35 years (76.5%, n = 423), with a predominance of the female sex (79.7%, n = 441). Nearly two-thirds (62.9%, n = 348) were single, and more than three-quarters (76.1%, n = 421) were university degree holders. More than half (56.2%, n = 311) were still students, and approximately 64.4% (n = 356) were earning less than 5,000 Saudi Arabian Riyals (SAR) per month. Respondents who were living in Makkah city constituted 48.1% (n = 266). Government hospitals were preferred by more than half of the participants (52.3%, n = 289), with 43.9% (n = 243) preferring clinics inside Makkah city. The most common vision-corrected device being used was eyeglasses, while the most commonly diagnosed eye disease was a refractive error (43.2%, n = 239). Approximately 23% (n = 126) of the patients had associated comorbidities, and the most commonly recorded were obesity (8.3%, n = 46) and diabetes mellitus (7.1%, n = 39) (Table 1).

**Table 1:** Sociodemographic characteristics and ophthalmologic problems of the study sample (n = 553) SAR: Saudi Arabian Riyal

Variables	N	(%)
Age group		
18 – 35 years	423	(76.5%)
36 – 65 years	121	(21.9%)
More than 65 years	9	(1.6%)
Gender		
Male	112	(20.3%)
Female	441	(79.7%)
Marital status		
Single	348	(62.9%)
Married	178	(32.2%)
Divorced or widowed	27	(4.9%)
Educational status		
High school and below	132	(23.9%)
University degree	421	(76.1%)
Occupational status		
Student	311	(56.2%)
Government employee	99	(17.9%)
Private employee	48	(8.7%)
Unemployed	95	(17.2%)
Monthly income (SAR)		
Less than 5,000	356	(64.4%)
5,000 – 10,000	106	(19.2%)
More than 10,000	91	(16.5%)
Residence area		
Inside Makkah city	266	(48.1%)
Other cities in the Makkah region	287	(51.9%)
Type of hospital usually visit		
Government	289	(52.3%)
Private	264	(47.7%)
Hospital location		
Inside Makkah city	243	(43.9%)
Other cities in the Makkah region	310	(56.1%)
Use of vision corrective device		
Not using	242	(43.8%)
Eyeglasses	211	(38.2%)
Contact lenses	16	(2.9%)
Both	84	(15.2%)
Eye diseases		
None	207	(37.4%)
Refractive errors	239	(43.2%)
Cataract	6	(1.1%)
Age-related macular degeneration	88	(15.9%)
Astigmatism	6	(1.1%)
Keratoconus	7	(1.3%)
Associated comorbidity		
Yes	126	(22.8%)
No	427	(77.2%)

The assessment of patient satisfaction regarding healthcare services in ophthalmology clinics was composed of seven domains. For the accessibility domain, suitable working hours at the ophthalmology clinics had the highest rate among the participants (mean score: 2.64). For the continuity items, most patients reported that the doctor's easy access to their medical records made them more satisfied with the services in these clinics (mean score: 2.70). Regarding the humanness domain, many patients acknowledged the respectful way that doctors treated them (mean score: 2.84). For the comprehensive domain, many patients voted that they received a comprehensive medical examination in ophthalmology clinics (mean score: 2.59). For the communication domain, most patients said that their doctors listened to them well while receiving their eye care (mean score: 2.82). A lot of the study participants reported that ophthalmologic specialists explained to them the reasons to do the tests and treatment adherence (mean score: 2.70). Finally, regarding the center satisfaction domain, many patients suggested that services provided at ophthalmologic clinics can be improved more (mean score: 2.72). The grand total mean ± SD for satisfaction was 88.3 ± 11.1. Accordingly, 75% (n = 415) of the patients were classified as satisfied, while 25% (n = 138) were dissatisfied (Table 2).

**Table 2:** Assessment of patients’ satisfaction with the healthcare services provided in ophthalmology clinics (n = 553) Response has a range from 1=disagree to 3=agree.

Satisfaction statement	Mean ± SD
Accessibility items	9.18 ± 1.42
Working hours at the clinic are suitable for all.	2.64 ± 0.59
The clinic gives me access to medical care at any time I need it.	2.43 ± 0.72
The time spent in the waiting room for a routine visit is very long.	2.20 ± 0.80
I find it difficult to get an appointment.	1.91 ± 0.84
Continuity items	7.03 ± 1.53
My doctor can easily access my medical records.	2.70 ± 0.54
I see the same doctor at each visit.	2.32 ± 0.82
The clinic will contact me if I don't come to the follow-up appointment.	2.01 ± 0.79
Humanness Items	16.1 ± 2.21
The doctors at the clinic treat me with respect.	2.84 ± 0.42
Nurses, laboratories, and other staff treat me well.	2.80 ± 0.45
The staff at the clinic keeps my health information confidential.	2.77 ± 0.46
Officials at the clinic listen to the complaints of the patients.	2.61 ± 0.60
The clinic's receptionist treats me well.	2.60 ± 0.62
The Health Center provides health services in emergency situations.	2.51 ± 0.59
Comprehensiveness Items	12.6 ± 2.31
The doctor provides me with a comprehensive medical examination when I need it.	2.59 ± 0.62
The results of laboratory tests are attached immediately to the file.	2.56 ± 0.57
The data in the medical file are comprehensive and accurate.	2.54 ± 0.61
The medical staff at the clinic are familiar with the latest medical developments.	2.48 ± 0.63
In each medical visit, they measure weight, height, blood pressure, and temperature.	2.40 ± 0.76
Communication Items	12.9 ± 1.59
The doctor listens to me well.	2.82 ± 0.46
The doctor does not answer all my questions.	2.82 ± 0.46
The doctor treated me in a friendly and very nice way.	2.79 ± 0.46
Time spent together with the doctor is enough.	2.67 ± 0.62
The doctor sometimes makes me feel like I'm an idiot.	1.76 ± 0.80
Education items	22.6 ± 4.57
The specialist explained to me the reason for doing the tests and treatment adherence.	2.70 ± 0.60
The specialist gave me enough information about my health.	2.69 ± 0.58
The language used in brochures is simple and easy to understand.	2.34 ± 0.65
The specialist shows his enthusiasm and interest in the sessions.	2.27 ± 0.72
Many brochures about common health problems are available in the clinic.	2.19 ± 0.73
The number of awareness programs that are held in the center is appropriate to the patient's needs.	2.17 ± 0.70
The center does not care to provide educational brochures to the patient.	2.14 ± 0.71
There is a diversity of educational resources (audio/visual).	2.16 ± 0.72
There is a place for educational sessions.	2.03 ± 0.71
There are educational films displayed in the waiting rooms.	1.92 ± 0.84
Clinic satisfaction items	7.87 ± 1.29
I think that the services provided at the ophthalmology clinic can be better than they are right now.	2.72 ± 0.53
Instruments and equipment in the ophthalmology clinic are working correctly.	2.69 ± 0.54
The ophthalmology clinic is always tidy.	2.46 ± 0.73
Total satisfaction score	88.3 ± 11.1
Level of satisfaction	N (%)
Satisfied	415 (75.0%)
Dissatisfied	138 (25.0%)

When conducting regression analysis, it was observed that compared to the younger age group, the odds of being satisfied with ophthalmology clinics were predicted to decrease by at least 44% among those aged more than 35 years (OR = 0.437; 95% CI = 0.257-0.743; p = 0.002). Patients who had been married were predicted to decrease the chance of being satisfied by at least 50% compared to patients who had never been married (OR = 0.538; 95% CI = 0.352-0.823; p = 0.004). Compared to students, patients who were currently employed were predicted to decrease the chance of being satisfied by at least 48% (OR = 0.481; 95% CI = 0.270-0.856; p = 0.013). We observed that people who had a higher monthly income had decreased odds of being satisfied by at least 58% compared to those who earned less monthly (OR = 0.583; 95% CI = 0.381-0.893; p = 0.013). In contrast, compared to patients with associated comorbidity, patients who have no comorbidity were predicted to have an increased chance of being satisfied by at least two-fold than those who had comorbidities (OR = 2.023; 95% CI = 1.199-3.413; p = 0.008) (Table 3).

**Table 3:** Factors associated with overall levels of patient satisfaction with healthcare services in ophthalmology clinics (n = 553) OR: odds ratio; CI: confidence interval; SAR: Saudi Arabian Riyal; **significant at p<0.05 level

Factor	Level of satisfaction		OR (95% CI)	P-value
	Satisfied N (%) (n=415)	Dissatisfied N (%) (n=138)		
Age group				
≤35 years	304 (73.3%)	119 (86.2%)		
>35 years	111 (26.7%)	19 (13.8%)	0.437 (0.257 – 0.743)	0.002 **
Gender				
Male	92 (22.2%)	20 (14.5%)		0.052
Female	323 (77.8%)	118 (85.5%)	1.680 (0.991 – 2.848)	
Marital status				
Never been married	247 (59.5%)	101 (73.2%)		
Been married	168 (40.5%)	37 (26.8%)	0.538 (0.352 – 0.823)	0.004 **
Educational level				
High school or below	104 (25.1%)	28 (20.3%)		0.255
University degree	311 (74.9%)	110 (79.7%)	1.313 (0.820 – 2.103)	
Occupational status				
Student	214 (51.6%)	97 (70.3%)		
Employed	123 (29.6%)	24 (17.4%)	0.481 (0.270-0.856)	0.013 **
Unemployed	78 (18.8%)	17 (12.3%)	1.117 (0.564 – 2.211)	0.751
Monthly income (SAR)				
<5,000	255 (61.4%)	101 (73.2%)		
≥5,000	160 (38.6%)	37 (26.8%)	0.583 (0.381 – 0.893)	0.013 **
Residence area				
Inside Makkah	193 (46.5%)	73 (52.9%)		0.193
Outside Makkah	222 (53.5%)	65 (47.1%)	0.774 (0.526 – 1.138)	
Type of hospital usually visit				
Government	212 (51.1%)	77 (55.8%)		0.337
Private	203 (48.9%)	61 (44.2%)	0.827 (0.561 – 1.218)	
Hospital location				
Inside Makkah	175 (42.2%)	68 (49.3%)		0.145
Outside Makkah	240 (57.8%)	70 (50.7%)	0.750 (0.510 – 1.104)	
Use of vision corrective device				
Yes	236 (56.9%)	75 (54.3%)		0.605
No	179 (43.1%)	63 (45.7%)	1.107 (0.751 – 1.631)	
Eye diseases				
Yes	261 (62.9%)	85 (61.6%)		0.785
No	154 (37.1%)	53 (38.4%)	1.056 (0.710 – 1.571)	
Associated comorbidity				
Yes	106 (25.5%)	20 (14.5%)		
No	309 (74.5%)	118 (85.5%)	2.023 (1.199 – 3.413)	0.008 **

## Discussion

This study was carried out to determine the satisfaction level of patients with the healthcare services that they received in the ophthalmology clinics in the Makkah region of Saudi Arabia. The study involved 553 patients who attended ophthalmology clinics during the time of data collection, and most of them (76.5%, n = 423) were between the ages of 18 and 35, which is like Khathlan's retrospective descriptive study in Saudi Arabia [18], which found that the majority of patients who visited the ophthalmology clinics in 2018 were between the ages of 21 and 40. Also, Bambamba et al. [19] conducted research to assess patient satisfaction with ophthalmology clinics in Mozambique, and they reported that 66.5% of their study sample was in the age group of 20-40 years old. This issue should be examined in further research to detect the common reasons for the increased frequency of ophthalmology clinic visits among young adults in comparison to the other age groups.

There was great satisfaction among patients with the healthcare services provided in ophthalmology clinics in the Makkah region of Saudi Arabia. Three-quarters (75%, n = 415) of this study subjects were considered satisfied, and the rest (25%, n = 138) were dissatisfied. This is consistent with the report of Alkhalaileh et al. [10]. According to their reports, the overall satisfaction of the patients visiting the outpatient eye clinic at Saint John Hospital was 63.9%. However, studies conducted in Nigeria [20] and Southwestern Ethiopia [21] documented relatively high satisfaction levels with the service provided by the ophthalmic clinics, accounting for 95.4% and 97.8%, respectively. The ages of the participants in the previously done two studies were above 30 years old, which is contradictory to that in our study; hence, this could be a source of the disparity between their and our studies' findings. Contradicting these reports, a study published in Vietnam [22] reported a lower satisfaction rate, as only less than half were satisfied with the services given by the eye care services, with only 6.8% considered very satisfied with all domains of care. The cause of this great difference in satisfaction levels between their study subjects and ours is related to the disparities in the economic status of both studies' settings, which may have an impact on the quality of healthcare services and the availability of the required resources.

Moreover, this study explored the fact that all domains of satisfaction had relatively higher mean scores, with an overall mean score (M± SD) of (88.3 ± 11.1). More than 75% of the patients were satisfied with the healthcare services in ophthalmology clinics in the Makkah region of Saudi Arabia. Considering this scenario, Khan et al. [11] also observed excellent satisfaction levels among patients visiting the ophthalmic clinic at the primary healthcare center in Al Ahsa district, Saudi Arabia.

More than 90% of the study subjects were satisfied with the way of communicating with their ophthalmologists, the convenience of attending the community eye care clinic, eye health education, the benefits of saving time and money, and their satisfaction with follow-up management. Undergoing minor surgeries also came back with good satisfaction among the study sample, as more than 80% were satisfied with the results of their ophthalmic operations. In Malaysia [15], service factors or tangible priorities were the highest in patient satisfaction, mainly in the form of “technical quality” and “accessibility and convenience,” but lower satisfaction was noted in terms of the service orientation of doctors, specifically the “time spent with the doctor,” “interpersonal manners,” and “communication” during consultations. However, in a study done in Nigeria [20], the investigators noted that despite the majority of respondents (50.8%) indicating that the waiting time for emergency treatment was good, the waiting time for performing an investigation and subsequently getting the result was reported as poor by 62.5% and very poor by 69%, respectively. Minimizing the waiting time for patients who need consultations will significantly improve satisfaction rates. Hence, the deterrence factors of satisfaction must be eliminated to improve patients' levels of satisfaction.

Data from this study indicate that being older, having been married, currently employed, having a higher economic status, and having comorbidities were the independent significant predictors of decreased satisfaction toward ophthalmology clinics. In a study by Ganasegeran et al. [15], there were also significant differences between the demographic variables and patient satisfaction, including gender, income level, and purpose of visit to the clinic, while according to Lorato et al. [23], being unmarried and having a higher monthly income were associated with patient satisfaction. However, Van Huy et al. [22] came up with conflicting results. Accordingly, they observed significant differences in patient satisfaction with eye care in terms of department, residence, and education but not in monthly income. Also, they discovered that female patients who came from rural and remote areas tended to exhibit better satisfaction levels than male patients living in urban areas. The variability in factors affecting patient satisfaction with healthcare services in ophthalmology clinics in different countries may be due to the differences in the sample characteristics and services provided in these countries.

Study limitations and strengths

There were some limitations in this study. First, the age distribution was not equal, which may not be representative of a true population and indicates a selection bias that may influence the satisfaction rates. In order to overcome this issue in the next studies, researchers should take age distribution into account while stratifying the sample into multiple age groups, each requiring a minimum number of participants to be met during data collection. Second, gender distribution was also not equally collected; thus, we cannot generalize the comparison of satisfaction between male and female patients. Third, online surveys may be prone to bias because respondents can answer according to their preferences, which may lead to false information in some cases. Finally, since the study is cross-sectional, it is prone to disadvantages, including cause-and-effect relationships and bias. Despite the previously mentioned limitations, this study gave a global view of what makes patients satisfied with the healthcare services provided in ophthalmology clinics. Additionally, the study highlighted an issue that was accidentally discovered and needs further investigation to prove or ignore, which is the higher frequency of young adults in ophthalmology clinics. Therefore, further research is recommended to explore the reasons.

## Conclusions

This study concludes that the frequency of patient satisfaction with the healthcare services provided in ophthalmology clinics in the Makkah region of Saudi Arabia was 75%. Suitable working hours at the ophthalmology clinics, continuity of care through easy accessibility of the ophthalmologist with their medical records, doctors professionalism, conducting comprehensive examinations, doctors listening to them during the visits, information received about their diseases, and management plans are all conditions that made patients satisfied with the healthcare services in ophthalmology clinics. According to the study, patient age, occupation, marital status, monthly income, and associated comorbidities are all factors that influence patient satisfaction with medical care in ophthalmology clinics. Further studies in the form of focus group discussions or directed interviews are recommended to give an in-depth view of the patients regarding the healthcare services provided in the ophthalmology clinics and other healthcare facilities to improve the quality of these services.
